# A Rare Presentation of Artery of Percheron Infarct: A Case Report

**DOI:** 10.7759/cureus.47548

**Published:** 2023-10-23

**Authors:** Muzamil Musa, Sondos K Khalil, Leena Saeed, Nawras Hatam Mahdy Al-tikrety, Motaz M Almahmood, Ahmed Alsayed, Salma Mustafa, Dalal Sibira

**Affiliations:** 1 Internal Medicine, Hamad Medical Corporation, Doha, QAT; 2 Internal Medicine, Hamad General Hospital, Doha, QAT; 3 Radiology, Hamad Medical Corporation, Doha, QAT

**Keywords:** percheron, stroke, brain, thalamus, midbrain, artery

## Abstract

The artery of Percheron (AOP), a variation of the thalamic vasculature, supplies both the thalamus and the midbrain. An infarct in this area is characterized by wide neurological abnormalities, the most common of which are altered mental state, decreased degree of consciousness, and memory impairment. AOP infarcts tend to be missed during the initial computed tomography (CT) scan. The number of reports on AOP infarction has been increasing, highlighting the range of clinical presentations and challenges that clinicians can face. This case study discusses a 58-year-old male patient who was diagnosed with stroke in AOP territory without any clear neurological symptoms, and it serves as a model for patients with similar conditions.

## Introduction

Variation in the anatomy of the human body is a well-known phenomenon described in the scientific literature. Such variation ultimately results in diversity in clinical presentations when exposed to an insult. The thalamic blood circulation is no exception to this. The thalami have a blood supply categorized into four territories: anterior, paramedian, posterior, and inferolateral [1-3]. The paramedian territories of the thalami are supplied by perforating thalamic arteries of the posterior circulation known as paramedian arteries [4]. The artery of Percheron (AOP) is a normal variant of the paramedian arteries. In this variant, there is only one AOP, which originates from the P1 segment of the posterior cerebral artery unilaterally and then bifurcates to supply the thalamus bilaterally. As a result, AOP damage will result in bilateral thalamic disease. This variation is found in between 4% and 12% of the population [3]. AOP infarction is thought to be relatively infrequent, accounting for 0.1%-2% of all ischemic strokes, according to stroke series studies [5]. We present the case of a 58-year-old man who was admitted to the hospital after experiencing intense tingling sensations in his tongue and blurred vision and was diagnosed with a bilateral thalamus stroke caused by AOP ischemia.

## Case presentation

A 58-year-old right-handed male with a past medical history of well-controlled asthma on inhalers was referred from a private hospital to our emergency department as a case of possible stroke. Upon questioning, he complained of tongue heaviness, bilateral blurring of vision, and dizziness for two days. He reported no ear pain, tinnitus, or hearing loss. Additionally, he denied experiencing any headache, loss of consciousness, speech disorder, swallowing problems, motor weakness, or involuntary movements. He reported no abnormal sensory disturbances in his upper or lower limbs and no difficulty controlling his sphincters. He had no history of trauma or neck manipulation, and he did not report chest pain, palpitation, shortness of breath, abdominal pain, change in bowel habits, or fever. He is a non-smoker but occasionally consumes alcohol on weekends.

On examination, he was afebrile (36.6°C), had a normal blood pressure of 130/73 mmHg, a respiratory rate of 17 breaths per minute, a pulse rate of 70 beats per minute, and maintained saturation in room air. He was conscious, alert, and oriented to time, place, and person, following commands. He had a Glasgow Coma score of 15/15 with no ptosis. Pupils were equal and reactive to light. Extraocular movements were normal and symmetrical, with no diplopia, nystagmus, or facial asymmetry noted. The tongue appeared normal with regular movements. There was no neck stiffness; normal muscle bulk with no apparent or elicited fasciculations or deformities; and normal tone, power, and reflexes in both upper and lower limbs. He had a mild loss of temperature sense in the upper limb bilaterally. Light touch, pain, a sense of position, and vibration were normal. The National Institutes of Health Stroke Scale (NIHSS) score was 1. Chest, pericardium, and abdominal examinations were normal. His electrocardiogram was within the normal limit.

**Figure 1:**
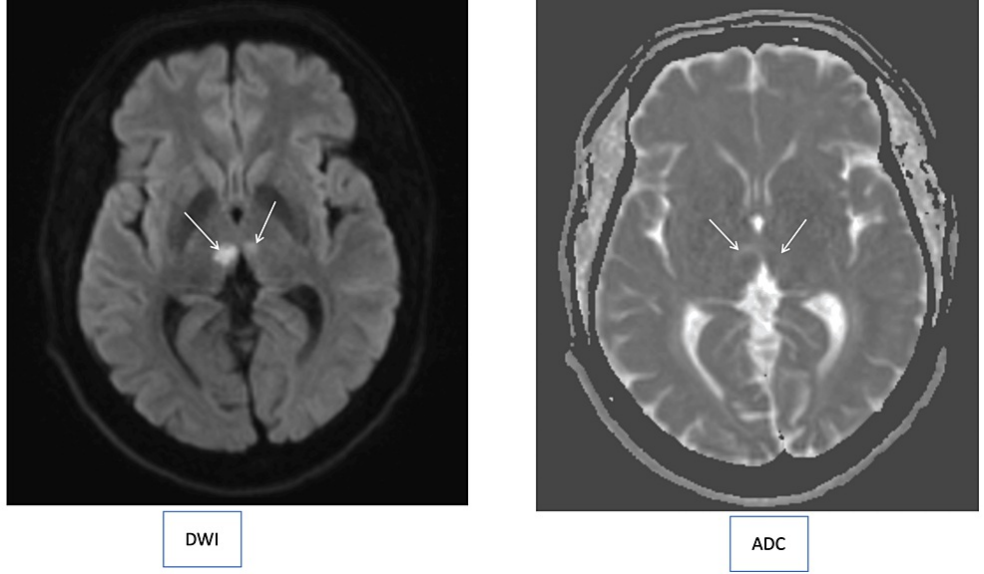
Bilateral medial thalamic area of diffusion restriction in DWI and ADC maps, larger on the right side compared to the left, consistent with acute ischemic lacunar infarcts along the AOP territory. DWI: diffusion-weighted imaging; ADC: apparent diffusion coefficient; AOP: artery of Percheron.

His cranial computed tomography (CT) in the private hospital reported a possible hypodensity in the right thalamus. A head MRI/magnetic resonance angiography (MRA) revealed bilateral medial thalamic areas of diffusion restriction larger on the right side, consistent with acute bilateral ischemic lacunar infarcts along the AOP territory, and the neck MRA was normal (Figure 1).

An echocardiogram showed evidence of a moderate patent foramen ovale (PFO). He was started on a dual antiplatelet regimen (aspirin 100 mg once daily (OD) and clopidogrel 75 mg) along with oral atorvastatin 80 mg OD, which was decreased to 40 mg OD the following day. His low-density lipoprotein (LDL) was 2.1 mmol/L (normal <2.59 mmol/L). He underwent a transesophageal echocardiogram (TEE), which revealed evidence of a large PFO measuring 12 mm by 4 mm. The etiology behind his stroke was attributed to a paradoxical embolus through his defect. His symptoms improved on the following day and completely resolved in two days with treatment, and he was discharged on the third day on dual antiplatelets and atorvastatin. The structural heart team was consulted, and they considered transcatheter PFO closure an outpatient procedure. He was sent home with a close follow-up and an event monitor. Four months later, he underwent PFO closure with a 30/30 mm Ceraflex device (Lifetech Scientific, Shenzhen, China). On the follow-up appointment, he reported a complete resolution of symptoms with no recurrence.

## Discussion

AOP is one of four morphological variations of the paramedian artery. Four anatomical variations in the vascular supply of the paramedian thalamus have been documented in the existing literature. Type I is characterized by the emergence of two paramedian arteries from the proximal segments of the posterior cerebral arteries. In Type IIa, two paramedian arteries originate from a single posterior cerebral artery. Type IIb, commonly referred to as the AOP, is marked by the single emergence of a paramedian artery from a solitary posterior cerebral artery, followed by its subsequent division to provide vascular support to both paramedian thalami. In Type III, there is communication between the two paramedian arteries that stem from both posterior cerebral arteries [6-8]. Notably, it is estimated that approximately one-third of the general population exhibits an anatomical variation involving the AOP [6-8]. The thalamus is a major gray matter component of the diencephalon that forms the lateral boundary of the third ventricle and contains more than 100 nuclei that serve as the final transmission point for afferent and efferent neurons [9]. The thalamus is supplied with blood by four branches of the posterior cerebral artery: the paramedian thalamic-subthalamic arteries, the thalamogeniculate arteries, and the posterior choroidal arteries.

The French neurologist Gerard Percheron described four arterial supply variations concerning the thalamus and the rostral midbrain. The third variant, specifically Type IIb, is characterized by the presence of the AOP. This particular variant is observed in a noteworthy portion of the population, with estimates suggesting its occurrence in up to 25% of individuals. Consequently, it plays a significant role in contributing to a considerable proportion of thalamic infarctions [10]. Ischemic infarction of the AOP occurs very rarely, accounting for 0.1%-2% of all strokes caused by ischemia, and ends up in paramedian thalami and mesencephalon infarction [11]. Drowsiness with a potential progression to a comatose state is often observed in conjunction with a range of associated clinical features. These features may encompass upward gaze palsy, abulia, and memory impairment, particularly when the involvement of the hippocampus is evident. Cognitive aberrations, such as emotional incontinence, hypersexuality, delusional thinking, and delirium, have also been documented. Additionally, in cases where the midbrain is affected, a distinct clinical syndrome known as thalamopeduncular syndrome becomes apparent, characterized by a triad of hemiplegia, cerebellar ataxia, and oculomotor palsy [10-12].

The accurate detection of an AOP occlusion is now a diagnostic challenge since the quality of treatment in managing an acute ischemic stroke depends on time, the anatomical location of the lesion, and contraindications to using thrombolytics [13]. When the condition is suspected, an MRI may assist in a rapid diagnosis. If the patient presents within the window period, treatment consists of thrombolysis. Aspirin and clopidogrel antiplatelet therapy should be initiated immediately, and risk factor modification should include smoking cessation, blood pressure control, and diabetes management.

Small artery disease (33%-38.9%), cardioembolic source (0%-22%), large vessel disease (13.2%-22.2%), and idiopathic causes (10%) are the main risk factors for AOP ischemia and thrombosis [12-15].

AOP stroke has an unclear long-term prognosis. In their follow-up, a group of 16 patients with paramedian infarction found chronic memory loss and lethargy in 13 patients and impairment of alertness in eight individuals [16].

## Conclusions

AOP infarcts can cause a variety of unusual signs and symptoms, which should be considered throughout the examination process. Infarction in this location is extremely varied in patient presentations and may often be devastating. As a result, earlier suspicion of AOP infarction may have resulted in earlier intervention and improved outcomes. We hope that this case presentation will raise awareness among physicians about these uncommon infarctions and their various presentations.
